# Estimated global overweight and obesity burden in pregnant women based on panel data model

**DOI:** 10.1371/journal.pone.0202183

**Published:** 2018-08-09

**Authors:** Cheng Chen, Xianglong Xu, Yan Yan

**Affiliations:** 1 Department of Epidemiology and Health Statistics, Xiangya School of Public Health, Central South University, Changsha, Hunan, China; 2 School of Public Health and Management, Chongqing Medical University, Chongqing, China; 3 Research Center for Medicine and Social Development, Chongqing Medical University, Chongqing, China; 4 Collaborative Innovation Center of Social Risks Governance in Health, Chongqing Medical University, Chongqing, China; Amsterdam UMC, location AMC, NETHERLANDS

## Abstract

**Objective:**

To estimate the global and country-level burden of overweight and obesity among pregnant women from 2005 to 2014.

**Methods:**

Publicly accessible country-level data were collected from *the World Health Organization*, *the World Bank* and *the Food* and *Agricultural Organization*. We estimated the number of overweight and obese pregnant women among 184 countries and determined the time-related trend from 2005 to 2014. Based on panel data model, we determined the effects of food energy supply, urbanization, gross national income and female employment on the number of overweight and obese pregnant women.

**Results:**

We estimated that 38.9 million overweight and obese pregnant women and 14.6 million obese pregnant women existed globally in 2014. In upper middle income countries and lower middle income countries, there were sharp increases in the number of overweight and obese pregnant women. In 2014, the percentage of female with overweight and obesity in India was 21.7%, and India had the largest number of overweight and obese pregnant women (4.3 million), which accounted for 11.1% in the world. In the United States of America, a third of women were obese, and the number of obese pregnant women was 1.1 million. In high income countries, caloric supply and urbanization were positively associated with the number of overweight and obese pregnant women. The percentage of employment in agriculture was inversely associated with the number of overweight and obese pregnant women, but only in upper middle income countries and lower middle income countries.

**Conclusion:**

The number of overweight and obese pregnant women has increased in high income and middle income countries. Environmental changes could lead to increased caloric supply and decreased energy expenditure among women. National and local governments should work together to create a healthy food environment.

## Introduction

Obesity is a growing public health hazard worldwide. The proportion of global adult women with overweight increased from 29.8% (29.3–30.2%) in 1980 to 38.0% (37.5–38.5%) in 2013, and the increasing trend was observed in both high income and middle income countries [1]. Among pregnant women, increased body mass index (BMI) was associated with numerous pregnancy related complications, including gestational diabetes mellitus (GDM), pregnancy hypertension and preeclampsia [2, 3]. Women with overweight or obesity involved a relatively high risk of severe maternal morbidity and mortality. Previous experts reported a odds ratio (OR) for severe maternal morbidity of 1.1 for women with obesity class 1 (BMI 30.0–34.9) compared with women with normal weight (BMI 18.5–24.9) [4]. The OR for obesity class 2 (BMI 35.0–39.8) was 1.2, and for obesity class 3 (BMI ≥40) was 1.4. Maternal obesity also increased perinatal mortality. A previous cohort study found that maternal obesity was associated with nearly 25% of stillbirth that occurred between 37 and 42 weeks’ gestation [5]. In addition, overweight and obesity were associated with elevated risks of fetal macrosomia, some birth defects, and metabolic disease of children [6,7].

Considering the affect of pregnancy overweight and obesity on mothers and infants, it is need to investigate the burden of overweight and obesity among pregnant women. Thirty-two percent of Swedish pregnant women were overweight or obese in 2008–2010 [8]. The prevalence of overweight among pregnant women in Iceland increased form 25.9% to 27.7% within nine years [9]. According to a retrospective cohort study in Canada, twenty-two percent of pregnant women were obese and 24% were overweight in 2004–2014 [10]. A recent meta analysis reported that the prevalence of maternal obesity in Africa ranged from 6.5% to 50.7% [11]. However, previous studies exploring the prevalence of overweight and obesity among pregnant women were limited by the focus on a single country. The global burden of overweight and obesity among pregnant women remained unclear.

Many previous studies explored obesity of pregnant women and its determinants. For low-income women, fast food intake can increase caloric supply, which is sufficient to explain the increase of BMI in pregnant women [12, 13]. Compared with metropolitan residents, rural residents were more likely to be overweight and obese in many countries, such as the United States of America and China [14, 15]. However, countries with high rates of urbanization usually have higher rates of obesity than those with low rates of urbanization. A previous system review found a consistent positive association between urbanization and obesity in many countries in Southeast Asia, and the association was greater in low gross national income (GNI) countries [16]. Experts argued that urbanization could tip the balance between energy intake and energy expenditure, namely decreases in physical activity and increases in the consumption of cheap fast food [17]. Urbanization is usually accompanied by the transformation of industrial structure. In middle income and low income countries, a growing number of female work in service sectors. Occupational physical activity is an important determinant of daily energy expenditure. A previous study found that women in the sedentary occupation group had a higher risk of obesity compared to those in the agricultural occupation group if they had no education [18].

Although *the World Health Organization* (WHO) and *the Global Burden of Disease study* (GBD) provided data of obesity and overweight data among adults [19], those data has not been fully used to explore the prevalence of obesity and overweight among pregnant women. It is needed to explore more evidence about overweight and obesity among pregnant women. Therefore, the objectives of this study were to (1) estimate the global and country-level number of overweight and obesity among pregnant women from 2005 to 2014; (2) identify relative contributions of economic development, caloric supply, urbanization and female employment to the number of overweight and obese pregnant women.

## Methods

### Data sources

We derived an estimate of the number of overweight and obese pregnant women using publicly accessible country-level estimates of the following parameters: total population [20], crude birth rate [21], estimated prevalence of overweight and obesity in female [22]. In each country, the estimated overweight and obesity prevalence rate in female (>18 years) was age-standardized. We collected overweight and obesity data of 195 countries, birth rate data of 255 countries and population data of 265 countries. We excluded countries with missing data, and data of 184 countries form 2005 to 2014 were used in the final study. Eleven countries with missing data were excluded, namely Cook Islands, Monaco, Nauru, Niue, Saint kitts and Nevis, San Marino, South Sudan, Sudan, Sudan (former), Tuvalu and Dominica.

To evaluate the contribution of energy intake to overweight and obesity, we collected data of the food balance sheets (FBS) from *the Food and Agricultural Organization* (FAO) [23]. The FBS data were compiled from national accounts of the supply and use of foods. The data provided a comprehensive picture of food consumption at country-level, and reflected the increasing trend of per capita caloric supply. The database of FBS were updated in 2017, and the latest data were food supply in 2013. To reflect the changes of social demographic and economic characteristics, we also collected urbanization data, GNI data, and employment data from *the World Bank* [24]. Urbanization was the percentage of population residing in urban areas in each country according to national definition. GNI per capita data were in current U.S. dollars, divided by the midyear population, and deflated base on consumer price indexes. The indicators of employment were the percentages of employment in different industries of all female employment, including employment in industries, employment in services and employment in agriculture. Those factors were most ubiquitous in country-level and associated with the energy balance. In July 2017, we collected data of 184 countries form 2005 to 2013.

### Estimating the burden of overweight and obesity in pregnant women

BMI is defined as the weight in Kilograms divided by the square of the height in meters (Kg/m^2^) [25]. According to data in the WHO, a BMI of 25.0 kg/m^2^ or more is classified as overweight and obesity, and a BMI of 30.0 kg/m^2^ or more is defined as obesity. The point estimated number of overweight pregnant women was obtained using the following formula:

Estimated number of overweight pregnant women = Total population×crude birth $\mathrm{rate}\times \frac{280}{365}\times \mathrm{estimated}$ prevalence of overweight in female.

We multiplied the total population by the crude birth rate, and then by the average gestational period (280 days), to calculate the number of pregnant days, per country. By dividing the number by 365 days, we estimated the number of women pregnant on any given days during the year. Finally, by multiplying this number by the overweight prevalence, we calculated a point estimate of the number of overweight and obese pregnant women. Similarly, the number of obese pregnant women was calculated using the same method. This formula was adapted form a previous study which provided a useful method to estimate the number of pregnant women [26]. Country-level pointed estimates were added together to generate the global estimates of the number of overweight and obese pregnant women. According to the 95% confidence intervals of the overweight and obesity data, sensitivity analyses were used to provided the upper and lower bounds of the estimate number of overweight and obese pregnant women.

### Data analysis

Panel data were often termed time series and cross section data[27]. Compared with singular time series or cross-sectional analysis, panel data carried more information about the heterogeneity of individuals. The general model of the panel data can be described as the following formula:

$$y_{it}=\alpha_{it}+\mu_{it}+\beta_{it}x_{it}$$

*y*_*it*_ refers to an explained variable and *x*_*it*_ is an explanatory variable. *i* = 1…N refers to the individual index. *t* = 1…T refers to the time index. *α*_*it*_ is the intercept and *μ*_*it*_ shows the error term with classic assumptions. *β*_*it*_ represents the coefficient of *x*_*it*_.

According to different interceptions, panel data model includes three kinds of model, namely random effects model, pooled effects model and fixed effects model. We used the *F* test to choose fixed or pooled effects specification. Then, we used the Hausman test to choose fixed or random effects specification[28]. We used multivariable panel data models, and adjusted beta coefficients were provided. Significance level was set as *p* <0.05, and the *p* value used a two sided test. Microsoft Excel and R software version 3.3.1. were used to analyse these data.

## Results

Countries were divided into four groups by *the World Bank*, namely high income countries (HICs), upper middle income countries (UMICs), lower middle income countries (LMICs) and low income countries (LICs). There were 52 HICs, 53 UMICs, 49 LMICs, and 30 LICs. We estimated that there were 38.9 million overweight and obese pregnant women and 14.6 million obese pregnant women in 2014 (Table 1). LMICs carried the greatest burden of overweight and obesity in pregnant women, and UMICs carried the greatest burden of obesity in pregnant women. The burden of obesity in pregnant women was lower in LICs than in other countries. Data of 184 countries was provided in S1 and S2 Figs.

**Table 1:** Total number of overweight and obese pregnant women and percentage of global burden by WHO region in 2014.

Income group	Number of overweight and obese pregnant women (BMI≥25)		Number of obese pregnant women (BMI>30)	
	Total	Percentage of global burden	Total	Percentage of global burden
High income	5275800	13.5%	2552100	17.5%
Upper middle income	13646600	35.0%	5507100	37.7%
Lower middle income	15237800	39.1%	5116400	35.0%
Low income	4786800	12.3%	1425400	9.8%
All countries combined	38947100	..	14601100	..

**Fig 1:**
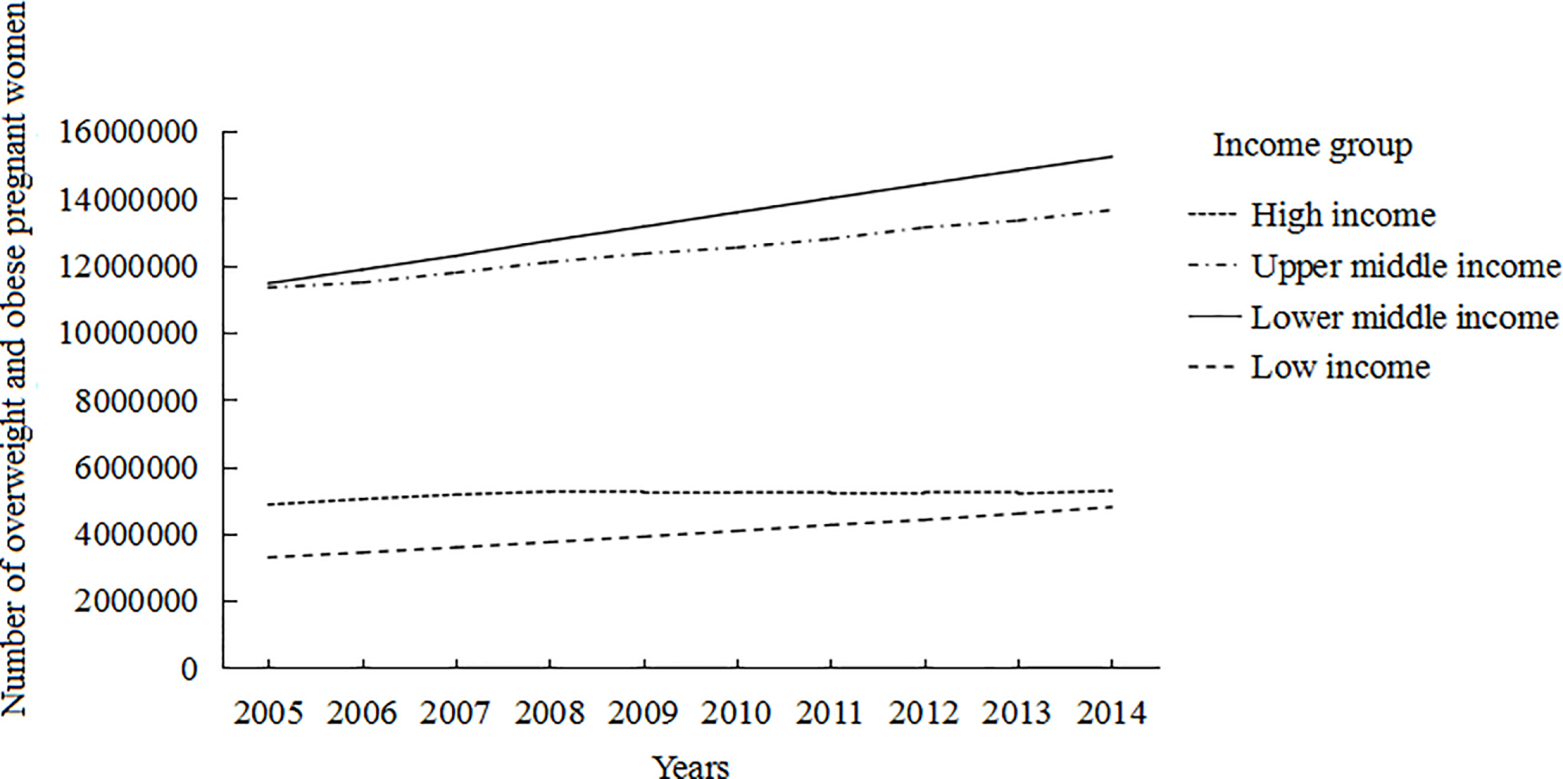
Number of overweight and obese (BMI>25) pregnant women by WHO region from 2005 to 2014.

**Fig 2:**
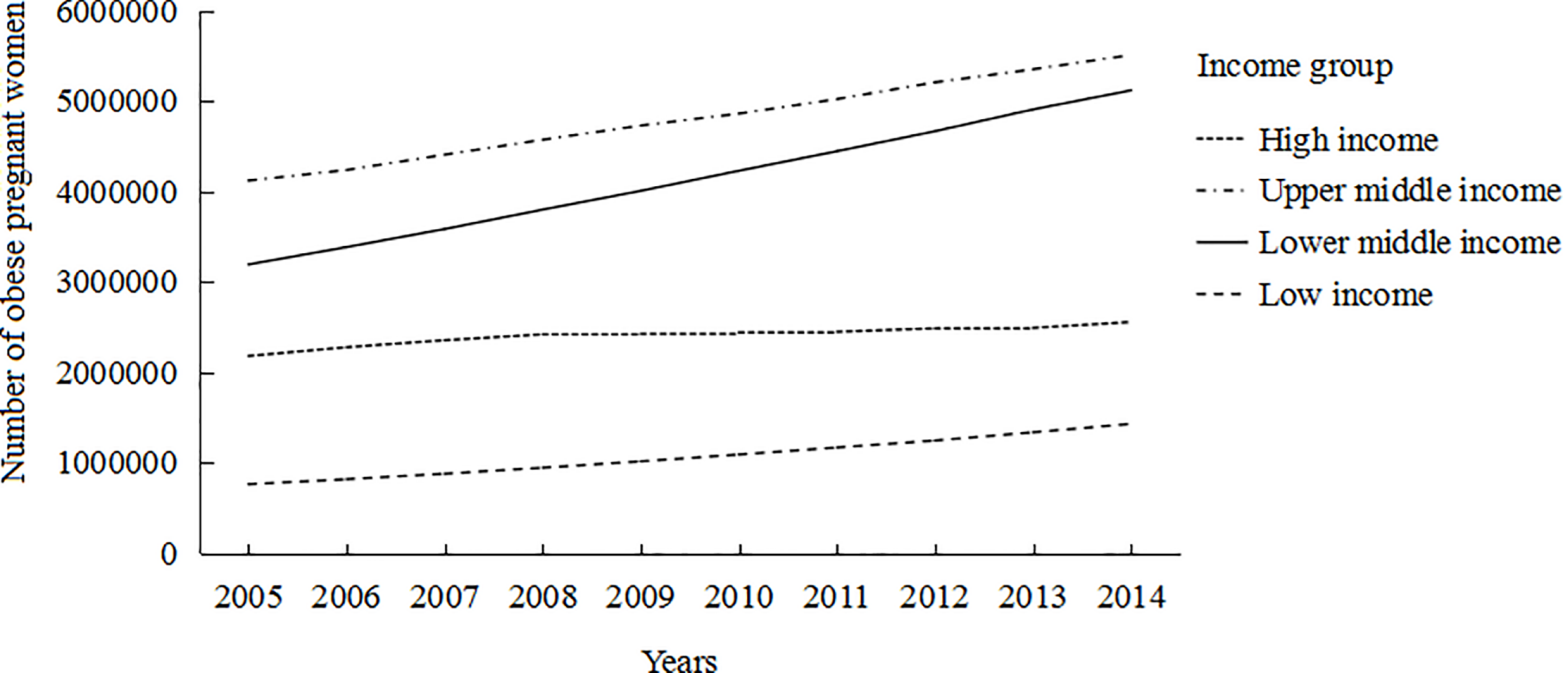
Number of obese pregnant women (BMI≥30) by WHO region from 2005 to 2014.

The increasing trends of overweight and obese pregnant women were observed in all income groups, but with different increasing patterns (Figs 1 and 2). LICs had the lowest number of overweight and obese pregnant women for many years. The number of overweight and obese pregnant women in UMICs and LMICs had a sharp increase. The number of obese pregnant women in UMICs was at a high level from 2005 to 2013. Although the number of obese pregnant women in LICs was at a low level, there was a increasing trend over that time period.

Estimates for 20 countries with the highest overweight and obesity burden in pregnant women were presented in Tables 2 and 3. In 2014, the percentage of female with overweight and obesity in India was 21.7%. India had the largest number of overweight and obese pregnant women (4.3 million), which accounted for the largest proportion (11.1%) in the world. The increases of overweight and obese pregnant women in some countries were more than 50%, such as Nigeria (55.4%), Democratic Republic of the Congo (53.4%) and United Republic of Tanzania (59.3%). For some countries with a high rate of overweight and obesity, the changes in ten years were small, such as United States of America (3.8%), Mexico (5.3%) and Turkey (5.9%). As the birth rate of Brazil decreased form 18.078 per 1000 people in 2005 to 14.727 per 1000 people in 2014, the number of overweight and obese pregnant women decreased by 1.7%. The United States of America had the largest number of obese pregnant women (1.07 million) in 2014. China also had 1.06 million obese pregnant women, and the number increased by 71.2% in ten years. For some countries with a high birth rate, the number of obese pregnant women was even doubled in ten years, such as Nigeria (96.9%), Democratic Republic of the Congo (102.2%), and United Republic of Tanzania (111.6%).

**Table 2:** Total number of overweight and obese (BMI>25kg/m^2^) pregnant women and rate of overweight among female for the 20 high overweight burden countries.

Country	Rate of overweight and obese among female [95% CI ]		Number of overweight and obese pregnant women [95% CI]		Changes in 10 years	Percentage of global burden in 2014
	2005	2014	2005	2014		
**India**^**c**^	16.6 [14.4–19.0]	21.7 [17.5–26.4]	3518500 [3090400–3997600]	4302000 [3509000–5180300]	22.3%	11.1%
**China**^**b**^	25.8 [22.9–28.8]	33.1 [27.4–39.2]	3199500 [2862200–3556700]	4285100 [3591200–5038500]	33.9%	11.0%
**Nigeria**^**c**^	30.3 [26.7–34.1]	39.6 [33.9–45.3]	1373600 [1217600–1539500]	2134900 [1845400–2431500]	55.4%	5.5%
**United States of America**^**a**^	58.4 [55.2–61.7]	62.9 [57.9–67.8]	1853400 [1756600–1952500]	1923400 [1780900–2065600]	3.8%	4.9%
**Egypt**^**c**^	64.2 [60.4–68.1]	70.2 [64.6–75.3]	921100 [869500–973800]	1340900 [1239500–1434500]	45.6%	3.5%
**Brazil**^**b**^	48.0 [44.1–51.9]	53.0 [46.9–59.3]	1254600 [1157400–1351000]	1233900 [1099800–1375200]	-1.7%	3.2%
**Mexico**^**b**^	60.8 [57.0–64.6]	65.2 [59.3–70.7]	1119400 [1053500–1185700]	1178300 [1076200–1273400]	5.3%	3.0%
**Indonesia**^**c**^	22.1 [18.8–25.6]	28.3 [22.9–34.2]	823700 [705200–947500]	1102300 [907500–1318900]	33.8%	2.8%
**Pakistan**^**c**^	21.0 [17.4–25.2]	26.0 [20.4–32.0]	741700 [621200–878000]	1079400 [859300–1315200]	45.5%	2.8%
**Russian Federation**^**b**^	53.5 [49.5–57.5]	54.8 [47.7–61.5]	600700 [558000–644200]	804100 [707500–897800]	33.8%	2.1%
**Turkey**^**b**^	63.5 [60.1–66.8]	68.4 [63.4–73.2]	645100 [612400–676800]	682900 [635500–727900]	5.9%	1.8%
**Islamic Republic of Iran**^**b**^	58.1 [55.0–61.2]	64.2 [58.7–69.4]	554800 [526600–583200]	675100 [619800–726000]	21.7%	1.7%
**Democratic Republic of the Congo**^**d**^	22.1 [17.5–27.3]	27.7 [21.0–35.2]	435200 [349300–533300]	667600 [514800–840900]	53.4%	1.7%
**Iraq**^**b**^	58.4 [53.4–63.2]	63.7 [57.1–70.0]	425200 [390400–458300]	596200 [537600–653600]	40.2%	1.5%
**Ethiopia**^**d**^	18.5 [15.0–22.3]	24.2 [18.5–30.2]	421700 [345100–503300]	583400 [452700–718600]	38.3%	1.5%
**South Africa**^**b**^	58.6 [54.4–62.9]	64.1 [58.5–69.4]	492800 [459100–527100]	550000 [504000–593300]	11.6%	1.4%
**United Republic of Tanzania**^**d**^	24.7 [21.1–28.5]	32.1 [26.6–37.9]	312500 [268900–358000]	497800 [417500–583800]	59.3%	1.3%
**Bangladesh**^**c**^	15.0 [12.2–18.1]	20.4 [15.8–25.8]	397200 [328600–474200]	492800 [387900–615000]	24.1%	1.3%
**Philippines**^**c**^	22.2 [18.9–25.7]	26.9 [21.3–32.9]	399500 [343700–458600]	481800 [386300–584200]	20.6%	1.2%
**Algeria**^**b**^	56.2 [51.5–60.9]	61.3 [54.8–67.4]	298600 [275000–322500]	445000 [400700–486900]	49.0%	1.1%

*Note*. CI: confidence intervals

^a^High income country

^b^Upper middle income country

^c^Lower middle income country

^d^Low income country.

**Table 3:** Total number of obese pregnant women,rate of obesity among female for the 20 high obesity burden countries.

Country	Rate of obesity among female [95% CI]		Number of obese pregnant women [95% CI]		Changes in 10 years	Percentage of global burden in 2014
	2005	2014	2005	2014		
**United States of America**^**a**^	30.2 [26.9–33.7]	34.9 [29.6–40.4]	958400 [860000–1064700]	1067200 [914000–1226200]	11.3%	7.3%
**China**^**b**^	5.0 [3.8–6.3]	8.2 [5.5–11.7]	620000 [479900–773800]	1061500 [731400–1491300]	71.2%	7.3%
**India**^**c**^	3.2 [2.5–4.1]	5.1 [3.4–7.2]	678200 [534100–862600]	1011000 [699800–1407500]	49.1%	6.9%
**Nigeria**^**c**^	9.3 [7.1–11.6]	15.4 [11.4–19.9]	421600 [326800–521300]	830200 [625900–1059400]	96.9%	5.7%
**Egypt**^**c**^	33.3 [29.1–37.7]	39.7 [33.2–46.1]	477700 [420400–537700]	758300 [640200–873500]	58.7%	5.2%
**Mexico**^**b**^	27.5 [23.8–31.5]	32.4 [26.4–38.7]	506300 [442600–575700]	585500 [483200–693100]	15.7%	4.0%
**Brazil**^**b**^	19.2 [16.2–22.3]	24.0 [19.1–29.4]	501800 [427600–577300]	558700 [450400–679500]	11.3%	3.8%
**Russian Federation**^**b**^	24.5 [21.0–28.3]	26.2 [20.1–32.7]	275100 [237600–316400]	384400 [298100–475400]	39.7%	2.6%
**Turkey**^**b**^	30.8 [27.3–34.3]	36.2 [31.0–41.8]	312900 [279100–346800]	361400 [313100–414700]	15.5%	2.5%
**Pakistan**^**c**^	5.7 [3.9–8.2]	8.2 [5.2–12.0]	201300 [141200–286400]	340400 [225000–489800]	69.1%	2.3%
**South Africa**^**b**^	32.7 [28.5–37.0]	38.1 [32.6–43.9]	182600 [133400–247100]	315500 [213400–449500]	18.9%	2.2%
**Indonesia**^**c**^	4.9 [3.5–6.7]	8.1 [5.3–11.7]	275000 [241900–309300]	326900 [281600–373500]	72.7%	2.2%
**Islamic Republic of Iran**^**b**^	24.8 [21.9–27.8]	29.7 [24.6–35.3]	236800 [210400–264100]	312300 [261700–368400]	31.9%	2.1%
**Iraq**^**b**^	26.8 [21.2–32.7]	32.1 [24.5–39.9]	195100 [156400–235600]	300400 [234200–368000]	54.0%	2.1%
**Algeria**^**b**^	25.3 [20.3–30.6]	29.7 [23.0–37.0]	134400 [109300–161400]	215600 [169000–265200]	60.4%	1.5%
**Saudi Arabia**^**b**^	35.6 [31.2–40.0]	40.7 [33.9–47.3]	90500 [57100–135100]	183100 [112300–281000]	23.1%	1.3%
**Democratic Republic of the Congo**^**d**^	4.6 [2.8–7.0]	7.6 [4.4–11.9]	157700 [139500–176300]	194200 [163700–224200]	102.2%	1.3%
**Argentina**^**b**^	24.5 [19.9–29.3]	30.1 [23.5–37.1]	138500 [113500–164100]	174100 [137900–213200]	25.7%	1.2%
**United Kingdom of Great Britain and Northern Ireland**^**a**^	23.4 [21.1–25.8]	28.4 [24.3–32.5]	130100 [117800–142900]	168900 [145900–192100]	29.8%	1.2%
**United Republic of Tanzania**^**d**^	6.2 [4.4–8.4]	10.7 [7.4–14.8]	78400 [56500–105100]	165900 [118000–227000]	111.6%	1.1%

*Note*. CI: confidence intervals

^a^High income country

^b^Upper middle income country

^c^Lower middle income country

^d^Low income country.

The changes in urbanization of different income groups were presented in S1 Table. In 2013, the urbanization rate in HICs and UMICs reached 80.6% and 63.7%, respectively. The urbanization rate in LMICs increased form 38.5% in 2005 to 41.5% in 2013. GNI of UMICs and LMICs increased by 50.2% and 54.0%, respectively (S2 Table). The Changes in caloric supply were presented in S3 Table. Caloric supply in HICs increased from 3221.0 kcal/capita/day in 2005 to 3263 kcal/capita/day in 2013. Caloric supply in LICs was 2324.4 kcal/capita/day in 2013, and increased by 5.8% (128 kcal/capita/day) in nine years. For many countries, the percentage of employment in agriculture decreased, while the percentage of employment in services increased (S4 Table). In 2013, the percentage of employment in agriculture was 1.8% in HICs, 9.3% in UMICs, 37.4% in LMICs, and 73.6% in LICs. For female in LMICs, the percentage of employment in services increased form 39.5% in 2005 to 50.7% in 2013.

As three indicators of employment were related to each other, we chose the percentage of employment in agriculture as the proxy of changes in occupational physical activity. According to the results of *F* test and Hausman test, random effects model was used for HICs, UMICs and LICs, and fixed effects model was used for LIMCs. For HICs, caloric supply (*p* = 0.001) and urbanization (*p* = 0.026) were positively associated with the number of overweight and obese pregnant women, and GNI (*p* = 0.004) was significantly associated with the number of obese pregnant women (Table 4). For UMICs and LMICs, the effect of caloric supply on the number of overweight and obese pregnant women was insignificant, and the percentage of employment in agriculture was inverse associated with the number of overweight and obese pregnant women. For LICs, urbanization (*p* = 0.005) and GNI (*p*<0.001)were significantly associated with the number of overweight and obese pregnant women.

**Table 4:** Factors associated with the number of overweight and obese pregnant women based on panel data model between 2005 and 2013.

Income group	Variable	BMI≥25			BMI>30		
		B	S.E.	p-value	B	S.E.	p-value
**Hig income**	Caloric supply ^a^	26.66	8.22	0.001	4.69	4.93	0.342
	Urbanization ^b^	1270.40	567.42	0.026	1432.70	337.98	<0.001
	Gross national income ^c^	0.06	0.13	0.628	0.22	0.08	0.004
	Employment in agriculture ^d^	-281.23	635.75	0.658	91.69	381.02	0.810
**Upper middle income**	Caloric supply	56.79	38.32	0.139	28.71	16.28	0.079
	Urbanization	8298.60	1736.20	<0.001	3606.40	704.35	<0.001
	Gross national income	3.73	1.54	0.016	2.81	0.66	<0.001
	Employment in agriculture	-2407.60	791.85	0.003	-1080.90	337.15	0.001
**Lower middle income**	Caloric supply	-5.98	56.58	0.916	-6.75	28.28	0.811
	Urbanization	6055.86	2755.61	0.029	2528.21	1377.31	0.067
	Gross national income	14.33	5.35	0.008	7.70	2.67	0.004
	Employment in agriculture	-4558.11	915.53	<0.001	-2437.66	457.60	0.000
**Low middle income**	Caloric supply	-52.11	26.67	0.052	-16.59	10.58	0.118
	Urbanization	3022.80	1074.76	0.005	1045.45	388.62	0.008
	Gross national income	82.61	10.70	<0.001	35.98	4.28	<0.001
	Employment in agriculture	665.17	451.05	0.142	267.44	178.75	0.136

Note.

^a^ kcal/capita/day

^b^ % of total population

^c^ current US$

^d^ % of female employment.

## Discussion

The large number of overweight and obese pregnant women was a huge burden on health care. This study estimated that nearly forty million pregnant women were overweight or obese in the world in 2014. More than 70% of overweight pregnant women occurred in UMICs and LMICs, owing to a large population and a high birth rate in those countries. The number of overweight and obese pregnant women increased rapidly in middle income countries from 2005 to 2014, especially in India, China and Nigeria. In many countries, more than half of women were overweight, and nearly a third of women were obese, such as Egypt, Turkey, Iran, and South Africa. More adverse maternal and fetal outcomes were observed in women with overweight and obesity. A previous study in Iranian found that pregnant women with obesity were 4 times more likely to develop gestational hypertension compared to those with normal weight [29]. Maternal obesity also increased the risk of fetal macrosomia, cardiac breaks, neural tube defects, and fetal death [30, 31, 32]. Health care providers should pay more attention to the adverse effects of obesity on maternal and fetal.

For HICs, the burden of overweight and obesity among pregnant women has been in a high level for many years.The increases of the number of overweight and obese pregnant women in UMICs and LMICs were faster than those in HICs. Those changes suggested a worldwide time-related phenomenon rather than a country-specific trend [33]. Previous studies found a slowdown in the increase rate of overweight and obesity in HICs, which provided some hope that the epidemic might had peaked in developed countries and that the populations in middle income countries might not reach the very high rates of over 40% [1]. However, considering the large population and the increasing rate of overweight in middle income countries, the burden of maternal overweight in those countries would be more serious in future.

Given that an increasing number of people lived in urban area, food environment and diseases of urban residents changed a lot [33, 34]. We found that urbanization was associated with the increasing number of overweight and obese pregnant women. City life can be more sedentary than rural life. A previous study found that BMI of urban residents was lower in countries with more land devoted to parks, which were sites for physical activity,walking and cycling [35]. A recent study in Seoul found that the number of sports facilities in urban were negatively associated with the probability of obesity [36]. City life also changes the availability of food, especially fast foods and energy-dense foods. Previous studies found that supermarkets were associated with a higher BMI among black adults [37]. The presence of convenience stores and fast food restaurants was a driver of weight excess, which usually offered energy-dense foods [38]. Although similar results shows that city life is associated with a higher risk of obesity than rural life, findings in the literature are not always consistent. A previous study found that the prevalence of obesity among women was higher in rural than in urban (33.4% vs 28.2%), and potential risk factors were lower leisure-time, intake of fiber and fruits and higher intake of sweetened beverages [39].

We found that food energy supply increased in many countries from 2005 to 2013. Previous studies in Venezuela and Ireland also reported a increasing trend of energy supply between 1961 and 2007 [40, 41]. We found that energy supply in HICs has been in a high level for many years. Compared with China and Japan, the consumption of total meat was higher in European Union, the United State of America and Canada [42]. This study found that caloric supply was a risk factor for the huge number of pregnant women with overweight in HICs, but not in other income group countries. A previous study about 69 countries also reported that the association between the change in food energy supply and the change in average body weight was significant for HICs [43]. For LICs, the increase of caloric supply might be a sign of improved nutrition.

This study found that GNI was positively associated with the number of obese pregnant women in all income groups. A previous study in thirty-three less developed countries found that GNI was positively associated with overweight among mothers [44]. Economic development can reduce food prices, especially prices of unhealthful foods. A previous study even reported that approximately 18% of growth in obesity could be attributed to relative food prices reduction between 1976 and 2001 [45]. This study found that the percentage of employment in agriculture was inversely associated with the number of overweight and obese pregnant women, but only in UMICs and LMICs. The main change in UMICs and LMICs was that a growing number of women were occupied in service sectors rather than in agriculture. Owing to the reduction in occupational physical activity, daily energy output among women has decreased by more than 100 kcal/day over the past 5 decades [46]. A study in Malaysia also reported that low occupational physical activity in middle-aged women was associated with higher risks of obesity and abdominal obesity [47].

Considering a growing number of overweight and obese pregnant women in both high income and middle income countries, health workers are faced with a huge challenge of reducing unfavorable pregnancy outcomes. According to the Institute of Medicine, the recommend GWG for overweight pregnant women is 7–11.9 kg and for obese pregnant women is 5–9 kg [48]. Dietary interventions and physical activity interventions were recommended to limit GWG and prevent GDM in overweight and obese pregnant women [49, 50, 51]. However, a randomised controlled trail in UK found that dietary and physical interventions in pregnant women with obesity were not adequate to prevent GDM or large-for-gestational-age infants, and a recent study in Australia also reported no significant differences in GDM between the behavioural nutrition intervention group and the control group after adjusting confounding factors [52,53]. From a public health perspective, it is a cost-effective strategy to control the prevalence of obesity among women of childbearing age. Women should be informed the potential risk of fast food and the importance of a normal weight for pregnant women. As the environment makes it easier to become overweight and obese, national and local governments should promote a health food environment, such as portion control, high calories food availability and media restrictions [54].

Some limitations exist in this study. Firstly, the data of overweight and obesity on reproductive age might be better than those across the whole age range. Unfortunately, data on reproductive age of many countries were not available form public accessible database. As the status of overweight and obesity can last for a long time, the present data can be used to approximate the number of overweight and obese pregnant women. Secondly, the definition of overweight and obesity is different in different regions, which can not be reflected in these international data. Overweight is defined as a BMI 25.0 to <30.0 kg/m^2^ by the WHO, and a BMI of 30.0 kg/m^2^ or more is defined as obesity. However, WHO Asia Pacific guidelines suggest that overweight is defined as BMI 23–27.49 kg/m^2^, and obesity is defined as BMI ≥ 27.5kg/m^2^ [55]. The overweight and obesity rate in some Asia countries would be underestimated using the former definition [55]. In the 2011 China Health and Nutrition Survey, obesity was defined as BMI ≥28.0 kg/m^2^, and the age-adjusted prevalence of obesity among women was 11.0%, which was higher than the prevalence provided by the WHO (7.1%) [56]. Thirdly, the level of urbanization, namely large metropolitan, small metropolitan and micropolitan, is also an important factor. Urbanization rate can not reflect these important information. Finally, this study used country as the unit of analysis in the panel data model, which might lead to ecological fallacy. We should not use country-level statistical findings to make inferences about the energy balance of individuals.

## Conclusion

There was a great increase of the number of overweight and obese pregnant women in both high income and middle income countries. Those data demonstrated that food energy supply, urbanization rate, GNI and employment in agriculture were associated with the burden of overweight and obese among pregnant women. In order to control obesity among pregnant women, national and local governments need to create a healthy food environment.

## Supporting information

S1 Fig The estimated number of overweight and obese pregnant women in 184 countries in 2014. (TIF)

S2 Fig The estimated number of obese pregnant women in 184 countries in 2014. (TIF)

S1 Table Changes of urban population in different income groups from 2005 to 2013. (DOC)

S2 Table Changes of gross national income in different income groups from 2005 to 2013. (DOC)

S3 Table Changes of food supply in different income groups from 2005 to 2013. (DOC)

S4 Table Employment in different industries of all female employment from 2005 to 2013. (DOC)
