# Supplementary figures and images for: Estimated global overweight and obesity burden in pregnant women based on panel data model
Source: PLoS One. 2018 Aug 9;13(8):e0202183. doi: 10.1371/journal.pone.0202183 (PMC6084991; doi:10.1371/journal.pone.0202183)

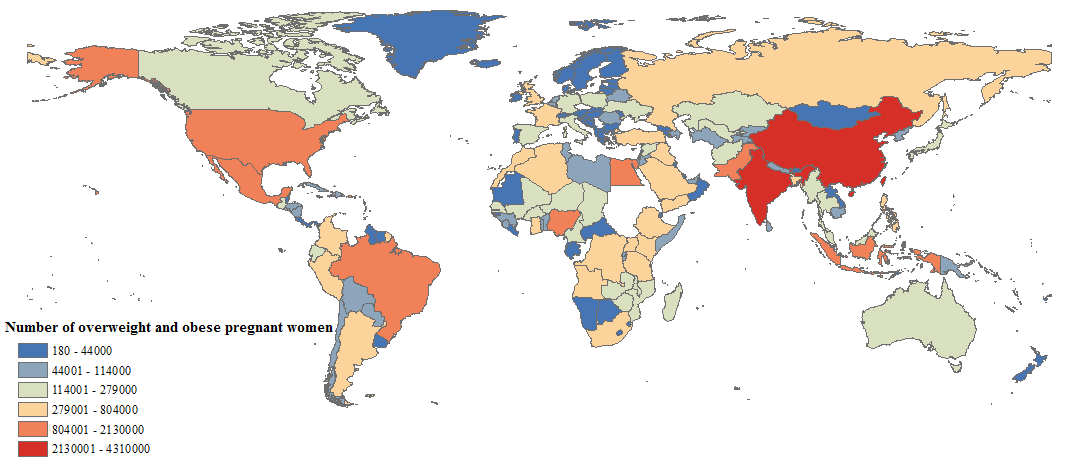

Supplement: S1 Fig — (TIF) [file pone.0202183.s001.tif]

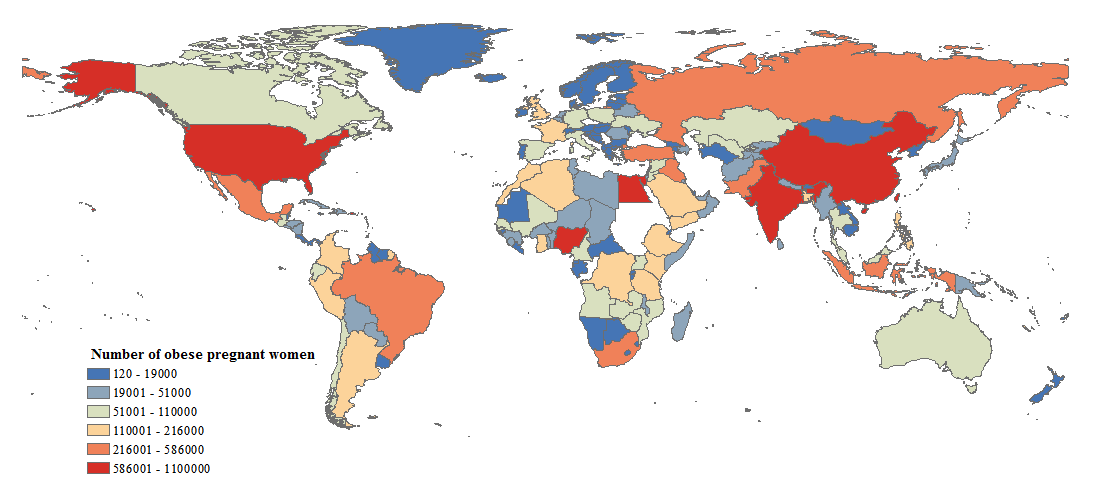

Supplement: S2 Fig — (TIF) [file pone.0202183.s002.tif]
