# Supplementary material for: Estimated global overweight and obesity burden in pregnant women based on panel data model
Source: PLoS One. 2018 Aug 9;13(8):e0202183. doi: 10.1371/journal.pone.0202183 (PMC6084991; doi:10.1371/journal.pone.0202183)
Supplement: S1 Table — (DOC) [file pone.0202183.s003.doc]

**S1 Table. Changes of urban population in different income groups form 2005 to 2013**

| **Income group** | **Urbanization in 2005 (%)** | **Urbanization in 2013 (%)** |
| --- | --- | --- |
|  | **P_50_ (P_25_-P_75_)** | **P_50_ (P_25_-P_75_)** |
| High income | 79.0 (67.4-87.0) | 80.6 (67.3-88.9) |
| Upper middle income | 62.1 (47.4-72.8) | 63.7 (53.0-75.9) |
| Lower middle income | 38.5 (26.7-54.0) | 41.5 (29.1-57.5) |
| Low income | 31.5 (20.2-38.0) | 33.8 (25.9-41.5) |
