# Supplementary material for: Estimated global overweight and obesity burden in pregnant women based on panel data model
Source: PLoS One. 2018 Aug 9;13(8):e0202183. doi: 10.1371/journal.pone.0202183 (PMC6084991; doi:10.1371/journal.pone.0202183)
Supplement: S2 Table — (DOC) [file pone.0202183.s004.doc]

**S2 Table. Changes of gross national income in different income groups from 2005 to 2013**

| **Income group** | **Gross national income (current US$)** | | **Change in 9 years** |
| --- | --- | --- | --- |
|  | **2005** | **2014** |  |
| High income | 32490.6±18752.4 | 40957.5±20430.4 | 26.1% |
| Upper middle income | 9281.7±3593.1 | 13940.0±4950.7 | 50.2% |
| Lower middle income | 3512.3±1655.3 | 5408.3±2428.7 | 54.0% |
| Low income | 1086.8±375.2 | 1473.0±506.8 | 35.5% |
