# Supplementary material for: Estimated global overweight and obesity burden in pregnant women based on panel data model
Source: PLoS One. 2018 Aug 9;13(8):e0202183. doi: 10.1371/journal.pone.0202183 (PMC6084991; doi:10.1371/journal.pone.0202183)
Supplement: S3 Table — (DOC) [file pone.0202183.s005.doc]

**S3 Table. Changes of food supply in different income groups from 2005 to 2013**

| **Income group** | **Caloric supply (kcal/capita/day)** | | **Change in 9 years** |
| --- | --- | --- | --- |
|  | **2005** | **2013** |  |
| High income | 3221.0±332.0 | 3263.6±290.8 | 1.3% |
| Upper middle income | 2791.7±327.8 | 2908.1±314.8 | 4.2% |
| Lower middle income | 2509.7±337.6 | 2642.2±336.6 | 5.3% |
| Low income | 2196.6±193.2 | 2324.4±251.6 | 5.8% |
