# Supplementary material for: Estimated global overweight and obesity burden in pregnant women based on panel data model
Source: PLoS One. 2018 Aug 9;13(8):e0202183. doi: 10.1371/journal.pone.0202183 (PMC6084991; doi:10.1371/journal.pone.0202183)
Supplement: S4 Table — (DOC) [file pone.0202183.s006.doc]

**S4 Table. Employment in different industries of all female employment from 2005 to 2013**

| **Income group** | **Female employment** | **2005 (%)** | **2013 (%)** |
| --- | --- | --- | --- |
|  |  | **P_50_ (P_25_-P_75_)** | **P_50_ (P_25_-P_75_)** |
| High income | Agriculture | 2.5 (1.1,4.9) | 1.8 (0.9,4.2) |
|  | Industry | 11.6 (9.8,17.1) | 9.5 (7.7,13.8) |
|  | Services | 85.7 (76.6,89.1) | 88.3 (81.9,91.3) |
| Upper middle income | Agriculture | 9.3 (4.7,31.3) | 9.3 (4.4,24.7) |
|  | Industry | 12.5 (8.9,20.4) | 10.9 (7.3,17.1) |
|  | Services | 69.2 (54.3,83.9) | 71.9 (61.3,84.3) |
| Lower middle income | Agriculture | 46.3 (33.3,65.9) | 37.4 (23.6,62.3) |
|  | Industry | 12.0 (3.9,17.2) | 11.0 (3.7,17.0) |
|  | Services | 39.5 (27.4,50.8) | 50.7 (34.3,59.7) |
| Low income | Agriculture | 78.5 (54.7,81.6) | 73.6 (57.4,82.5) |
|  | Industry | 2.5 (1.1,6.8) | 3.2 (1.5,6.4) |
|  | Services | 19.3 (16.6,43.5) | 21.2 (16.8,27.4) |
